# What would PCR assessment change in the management of fevers in a malaria endemic area? A school-based study in Benin in children with and without fever

**DOI:** 10.1186/1475-2875-9-224

**Published:** 2010-08-06

**Authors:** Jean-François Faucher, Agnès Aubouy, Todoégnon Béhéton, Patrick Makoutode, Grace Abiou, Justin Doritchamou, Pascal Houzé, Edgard Ouendo, Philippe Deloron, Michel Cot

**Affiliations:** 1 Institut de Recherche pour le Développement (IRD); UMR 216: Mother and Child face with Tropical infections Research Unit, Paris, France; 2 Departement of Infectious Diseases, Besançon University Medical Center, 25030 Besançon cedex, France; 3 Regional Public Health Institute of Ouidah, 01BP 918 Cotonou, Benin; 4 Faculty of Health Sciences, Department of Parasitology, Champ de Foire, 01BP 188 Cotonou, Benin; 5 Saint-Louis Hospital biochemistry laboratory, 1 avenue Claude-Vellefaux, 75010 Paris, France; 6 Université Paris Descartes, Faculté de Pharmacie, 4 avenue de l'Observatoire, 75006 Paris, France

## Abstract

**Background:**

A recent school-based study in Benin showed that applying a policy of anti-malarial prescriptions restricted to parasitologically-confirmed cases on the management of fever is safe and feasible. Additional PCR data were analysed in order to touch patho-physiological issues, such as the usefulness of PCR in the management of malaria in an endemic area or the triggering of a malaria attack in children with submicroscopic malaria.

**Methods:**

PCR data were prospectively collected in the setting of an exposed (with fever)/non exposed (without fever) study design. All children had a negative malaria rapid diagnostic test (RDT) at baseline, were followed up to day 14 and did not receive drugs with anti-malarial activity. The index group was defined by children with fever at baseline and the control group by children without fever at baseline. Children with submicroscopic malaria in these two groups were defined by a positive PCR at baseline.

**Results:**

PCR was positive in 66 (27%) children of the index group and in 104 (44%) children of the control group respectively. The only significant factor positively related to PCR positivity at baseline was the clinical status (control group). When definition of malaria attacks included PCR results, no difference of malaria incidence was observed between the index and control groups, neither in the whole cohort, nor in children with submicroscopic malaria. The rate of undiagnosed malaria at baseline was estimated to 3.7% at baseline in the index group.

**Conclusions:**

Treating all children with fever and a positive PCR would have led to a significant increase of anti-malarial consumption, with few benefits in terms of clinical events. Non malarial fevers do not or do not frequently trigger malaria attacks in children with submicroscopic malaria.

## Background

Restricting anti-malarials to parasitologically-confirmed cases has been recommended in many countries for children of five years of age or above. Discrepancies between laboratory results and anti-malarial prescriptions show that healthcare providers may not trust blood smear results in routine practice [1-3]. Providing that no diagnostic tool is perfect, a school-based prospective study (with an exposed to fever, not exposed to fever study design) was recently performed in Benin and found a similar incidence of malaria between groups in children with a negative malaria rapid diagnostic test at baseline. It was concluded that, at the scale of a health care facility, applying recent (anti-malarial prescriptions restricted to parasitologically confirmed cases) policies on the management of fever is safe and feasible in children aged of five years and above [4].

However, submicroscopic malaria is frequent in endemic areas, and no study yet has dissected the consequences of applying the algorithm of management of fever by asking the following questions: what would be the consequences in many endemic areas of treating with anti-malarials all febrile children with a malarial infection, as detected by PCR? and are children with submicroscopic malarial infections prone to develop malaria attacks soon after the occurrence of a non malarial fever?

Thus, the results of our recent study were re-analysed in the light of the PCR results collected during this trial.

## Methods

The data were prospectively collected through an exposed (with fever)/non exposed (without fever) study design. This study was performed from February through June 2008 in four schools, located at Allada, Southern-Benin, where malaria transmission is intense and perennial [5]. Altogether, the four study schools received about 2600 schoolchildren of 5 years-old and above. All children had a negative HRP2-based malaria rapid diagnostic test (RDT) at baseline, were followed up to Day 14, and did not receive drugs with anti-malarial activity. Performance of study methods were previously detailed [4]. The index group was defined by children with fever at baseline, and the control group by children without fever at baseline.

The primary objective of the study was to assess whether applying the algorithm of management of fevers in a school setting is consistent with an adequate management of fevers, i.e. it does not lead to a high number of undiagnosed (and thus untreated) malaria attacks, and by comparing the incidence of malaria attacks between the two groups (with and without fever) of children.

The secondary objective was to compare malaria incidence between children with submicroscopic malaria during the follow-up in the index group and the control group, respectively. Children with submicroscopic malaria in these two groups were defined by the detection of *Plasmodium falciparum *infection at enrolment, using the most sensitive diagnostic tool currently available (PCR). Based on one study from the literature, it was estimated that about 15% the proportion of children attending for fever with a negative RDT and a positive PCR for malaria [6], and it was admitted that this proportion would be roughly the same in apparently healthy children. Twenty-eight children with submicroscopic malaria in each group (corresponding to 187 children in each of the index and control groups) are needed to detect an 8% difference in proportion between the two groups (α = 0.05 and β = 0.8). Assuming a maximum of 5% in the dropout rate, it was planned to include a minimum of 200 children in each group.

The number of undiagnosed *P. falciparum *malaria at enrolment was estimated, using data collected both at enrolment and during the follow-up. At enrolment, all cases with *P. falciparum *parasitaemia > 1,000/μL were considered. During the follow-up an undiagnosed *P. falciparum *malaria at enrolment was defined as 1) at least one test (including PCR) positive at enrolment, 2) fever occurring during follow-up 3) at least one test (including PCR) positive for a *P. falciparum *infection at the time of fever.

### Laboratory analysis

DNA was prepared from blood collected at day 0 and on the last day of follow-up, as well as at occurrence of fever during follow-up. Blood collected on Whatman 3 MM filter paper was dried and conserved at room temperature until extraction. DNA was prepared by Chelex extraction, as described and subjected to a single PCR amplification protocol using primers specific for the small subunit ribosomal DNA (ssrDNA-PCR) as follows: rfal1 5'-TTAAACTggTTTgggAAAACCAAATATATT, rfal2 5'-ACACAATgAACTCAATCATgACTACCCgTC [7,8].

### Statistics

Parametric tests (Pearson's chi-square and chi-square for the comparison of crude rates [9]) and non-parametric (Fisher) tests were used. A logistic regression on PCR positivity at enrolment was performed, taking into account variables likely to influence the malarial status: gender, age, school, clinical status, declared bed net use.

## Results

Baseline data are presented in Table 1. Half of the children in the index group (124/242) had a temperature at or above 37.8°C and 29% (71/242) had a temperature at or above 38°C.

**Table 1:** Characteristics of children, matched on gender, age, week of inclusion, and rapid diagnostic test status.

Children characteristics	IG	CG	p value
Sex, no. male/female	104/138	105/137	0.93
Age, years; n = 242	9.1 (2.5)	9.1 (2.5)	0.95
Body temperature, °C; n = 242	37.7 (0.7)	37.1 (0.3)	< 10^-5^
Positive blood smear, day 0	12 (including 1 *P. malariae*); n = 242	3 (including 1 *P. malariae*); n = 236	0.02
Positive PCR, day 0	66; n = 242	104; n = 238	0.001
Bed net use	54; n = 234	46; n = 238	0.32
Chloroquine detection day 0	13; n = 100	6; n = 99	0.1
Chloroquine detection day 14	7; n = 98	8; n = 98	0.79
Quinine detection day 0	1; n = 100	0; n = 99	_
Quinine detection day 14	0; n = 99	0; n = 98	_

Quantitative data are mean (SD). SD: Standard deviation; IG: index group; CG: control group.

In the index group, PCR was positive in 66 (27%) children, including the 11 children with a blood smear positive for *P. falciparum*. In the control group, blood smears were positive in three children (two *P. falciparum *and one *Plasmodium malariae*); PCR was positive in 104 (44%) children. Results of multivariate analysis on PCR positivity are shown in Table 2. The only significant factor positively related to PCR positivity at baseline was the clinical status (control group), as the use of bed nets became marginally significant (in decreasing the risk of PCR positivity) after controlling for cofactors.

**Table 2:** Logistic regression analysis of factors possibly associated with PCR positivity, Allada, Benin, 2008.

	Univariate analysis		Multivariate analysis	
		
Variable	No pcr +, proportion (%)	Crude OR (95% CI)	Adjusted OR (95% CI)	*P*
Gender				
F	94/274 (34)	1.00	*1.00*	
M	76/206 (37)	1.12 [0.77-1.63]	*1.12 [0.77-1.63]*	*0.69*

Age*	-	-	-	*0.80*

School				
Allomey (1)	66/162 (40)	1.00	*1.00*	
Centre (2)	49/158 (31)	0.65 [0.41-1.04]	*0.70 [0.42-1.15]*	*0.16*
Dankoli (3)	14/37 (38)	0.89 [0.42-1.85]	*1.07 [0.50-2.28]*	*0.87*
Dogoudo (4)	40/122 (33)	0.71 [0.43-1.16]	*0.71 [0.42-1.22]*	*0.22*

Clinical status				
Control	104/238 (44)	1.00	1.00	
Index	66/242 (27)	0.48 [0.33-0.71]	0.42 [0.28-0.63]	< 10^-3^

Bednet use				
No	139/368 (38)	1.00	*1.00*	
Yes	27/100 (27)	0.61 [0.37-0.99]	*0.65 [0.38-1.10]*	*0.09*

* Not applicable, continuous variable

### Follow-up of index group children

Children of the index group were followed-up a total of 465 person-weeks (Table 3). When taking into account cases of fever with any positive malaria diagnostic test, eight malaria attacks were observed during follow-up.

**Table 3:** Malaria cases observed during follow-up in the whole cohort

Case definition (case numbers)	IG (242 patients)	CG (242 children)	p value
Fever + RDT	5	7	0.56
Fever + blood smear	6	5	0.76
Fever + PCR	7	10	0.46
Fever + any positive test	8	11	0.48

IG: index group; CG: control group.

Among the 66 children who had a positive PCR at baseline, eight presented with a malaria attack (fever with any positive malaria method) during the follow-up. In 58 cases, no fever occurred in the follow-up (including one case with a parasite density > 1,000/μL at enrolment).

There were two cases of undiagnosed *P. falciparum *malaria at enrolment, based on baseline criteria (*P. falciparum *parasitaemia > 1,000/μL) and eight cases based on follow-up data. The total number of undiagnosed *P. falciparum *malaria at enrolment was estimated to nine (one overlap) cases (3.7%).

### Follow-up of control group children

Children of the control group were followed-up a total of 477 person-weeks (Table 3). Eleven cases of fever related to malaria (fever with any positive parasitological test) were observed during follow-up. Nine of these 11 children presented with PCR positive at enrolment, while blood smear was negative in all. One malaria attack, as detected by RDT was not confirmed, neither by microscopic examination nor by PCR. The clinical picture in this case was stomatitis.

Among the 104 children who had a positive PCR at baseline, nine presented with a malaria attack (fever with any positive malaria method) during the follow-up. In 95 cases, no fever occurred in the follow-up. Incidence of malaria in children at risk for malaria (Table 4): There was no statistical difference, with any case definition, in terms of malaria incidence between the two groups.

**Table 4:** Malaria incidence (per week of follow-up) in children with submicroscopic malaria at baseline.

Case definition (case numbers)	IC (66 patients)	CG (104 children)	Chi-square	p value
Fever + RDT (IG: 5, CG: 5)	0.04065	0.02463	0.64	0.42
Fever + blood smear (IG: 6, CG: 4)	0.04878	0.0197	2.11	0.15
Fever + PCR (IG: 7, CG: 8)	0.05691	0.0394	0.51	0.48
Fever + any positive test (IG: 8, CG: 9)	0.06504	0.04433	1.02	0.31

Total follow-up in children at risk for malaria: 123 weeks in the IG and 203 weeks in the CG.

Chi square for the comparison of crude rates; IG: index group; CG: control group.

## Discussion

Many children with a negative RDT (nearly all of them had a negative blood smear as well) had a very low parasitaemia, in both groups. The concomitance of a negative RDT and a positive PCR is a proxy for submicroscopic malaria.

A recent study performed in Italy in African migrants found a high discrepancy between malaria blood smears and PCR data [10]. Submicroscopic malaria is a part of the burden of malaria in endemic areas, and may also have consequences in terms of malaria transmission. Overall, it has been estimated in endemic areas that prevalence of infection detected by microscopy was, on average, 49.2% lower than that detected by PCR [11]. The extent of discrepancies between microscopy and PCR results may reflect the magnitude of endemicity: the greater is endemicity, the lower is the proportion of infections undetected by microscopy [11]. In non-endemic areas, detection of a submicroscopic malaria in a feverish patient would certainly lead to an anti-malarial treatment. The question whether a more sensitive assay (PCR) than blood smears or RDTs would add to health care quality in the management of non malarial (based on RDT results) fevers in endemic areas is therefore an issue. In the study setting, treating all PCR positive children at baseline in the index group would have resulted into 66 additional malaria treatments, and might have avoided a maximum of eight malaria cases. In other terms, even though many RDT negative children may be infected with *P. falciparum*, treating them would be of poor benefit in terms of clinical events. These findings should not be extrapolated to the management of fever in younger children, who are less immune than children of this study population. Similarly, these findings may not be representative of school children from low transmission intensity areas who have developed very few functional clinical immunity. Similar follow-up type study should be repeated in different population and epidemiological settings in order to explore more extensively the implications of RDT treatment strategies.

Most children in the index group had another infection than malaria at baseline. The transient immunosuppression related to many infectious diseases might trigger malaria attacks in children with submicroscopic malaria at enrolment. The proportion of children who had malaria during the follow-up was 8% in children with a positive PCR at enrolment in the index group, while this proportion was 2% in the entire index group, and 4% in children with a positive PCR at enrolment in the control group. This suggests that non-malarial infections do not or do not frequently trigger malaria attacks in children with submicroscopic malaria.

The baseline status of the two groups was the negativity of RDT. Interestingly, microscopically detectable parasitaemia at baseline, although rare in both groups, was more frequent in the index group, while PCR detection at baseline was markedly more frequent in the control group. Differences in terms of self-medication can hardly explain these parasitological differences between groups, and the only factor related to PCR positivity was the clinical status at baseline. Because chloroquine and quinine were the most commonly drugs used for self-medication in the study area, they were chosen as markers of self-medication, though other anti-malarials like SP, were widely available at the time of the study. The higher rate of asymptomatic parasitaemia (as detected with PCR) in the control group may indicate that these children are less prone to develop symptomatic malaria and why not symptomatic infections in general.

The rate of estimated undiagnosed malaria attacks at enrolment was low. Baseline parasitological data may be used to estimate undiagnosed malaria attacks, but asymptomatic parasitaemia is a common status in the study population. Therefore, in an attempt to define the rate of undiagnosed malaria attacks at enrolment a combination of enrolment and follow-up data were used. It was postulated that undiagnosed malaria cases at baseline would lead in most cases to recurrence/persistence of fever concomitant to a positive malaria diagnostic test in the index group during follow-up. This definition has at least two main limitations: 1) it does not take into account that malaria may spontaneously heal in this population, and thus may lead to underestimate the "true rate"; 2) the use of a very sensitive diagnostic tool like PCR may lead to overestimate the "true rate" of undiagnosed malaria attacks.

## Conclusions

Treating all children infected with *P. falciparum *in this population would have led to a significant increase of anti-malarial consumption, with few benefits in terms of clinical events. Non-malarial fevers do not, or do not frequently, trigger malaria attacks in children with a submicroscopic infection.

## Competing interests

The authors declare that they have no competing interests.

## Authors' contributions

JFF designed the study, led field work interpreted the results and drafted the manuscript. PM, GA and TB participated in data collection. GA contributed with data collection in the field. AA, JD performed molecular assays. EO designed the study. PH performed pharmacology assays. PD designed the study and critically read the manuscript, MC designed the study, performed the statistical analyses and critically read the manuscript. All authors read and approved the manuscript.
